# Supplementary material for: Protocol for the development of a core outcome set for respectful maternal and newborn care in a low-middle income setting
Source: PLoS One. 2025 Apr 9;20(4):e0319419. doi: 10.1371/journal.pone.0319419 (PMC11981208; doi:10.1371/journal.pone.0319419)
Supplement: S1 Checklist — (DOCX) [file pone.0319419.s001.docx]

COS-STAP Checklist

| **SECTION/TOPIC** | **ITEM No.** | **CHECKLIST ITEM** | **Page No.** |  |
| --- | --- | --- | --- | --- |
| **TITLE/ABSTRACT** |  |  |  |  |
| Title  Abstract | 1a | Identify in the title that the paper describes the protocol for the planned development of a COS | 1 |  |
|  | 1b | Provide a structured abstract | 2 |  |
| **INTRODUCTION** |  |  |  |  |
| Background and objectives | 2a | Describe the background and explain the rationale for developing the COS, and identify the reasons why a COS is needed and the potential barriers to its implementation | 3-4 |  |
|  | 2b | Describe the specific objectives with reference to developing a COS | 4 |  |
| Scope | 3a | Describe the health condition(s) and population(s) that will be covered by the COS | 5 |  |
|  | 3b | Describe the intervention(s) that will be covered by the COS | 5 |  |
|  | 3c | Describe the context of use for which the COS is to be applied | 5 |  |
|  |  |  |  |  |
| **METHODS** |  |  |  |  |
| Stakeholders | 4 | Describe the stakeholder groups to be involved in the COS development process, rationale for their involvement, and a description of how the individuals will be identified | 8-9 |  |
| Information sources | 5a | Describe the information sources that will be used to identify the list of outcomes. Outline the methods or reference other protocols/papers | 6-8 |  |
|  | 5b | Describe how outcomes may be dropped/combined; with reasons | 7-8 |  |
| Consensus process | 6 | Describe the plans for how the consensus process will be undertaken | 10-12 |  |
| Consensus definition | 7a | Describe the consensus definition | 11 |  |
|  | 7b | Describe the procedure for determining how outcomes will be added/combined/dropped from consideration during the consensus process | 10 |  |
| **ANALYSIS** |  |  |  |  |
| Outcome scoring/feedback | 8 | Describe how outcomes will be scored and summarised, describe how participants will receive feedback during the consensus process | 10-11 |  |
| Missing data | 9 | Describe how missing data will be handled during the consensus process | N/A |  |
| **ETHICS/DISSEMINATION** | | |  | |
| Ethics approval / informed consent | 10 | Describe any plans for obtaining research ethics committee / institutional review board approval in relation to the consensus process and describe how informed consent will be obtained (if relevant) | 12 |  |
| Dissemination | 11 | Describe any plans to communicate the results to study participants and COS users, inclusive of methods and timing of dissemination. | 13 |  |
| **ADMINISTRATIVE INFORMATION** | | |  | |
| Funders | 12 | Describe sources of funding; role of funders | 13 |  |
| Conflicts of interest | 13 | Describe any potential conflicts of interest within the study team and how these will be managed | 13 |  |
