# Supplementary material for: Protocol for the development of a core outcome set for respectful maternal and newborn care in a low-middle income setting
Source: PLoS One. 2025 Apr 9;20(4):e0319419. doi: 10.1371/journal.pone.0319419 (PMC11981208; doi:10.1371/journal.pone.0319419)
Supplement: Attachment — Submitted filename: Response to Reviewers.docx [file pone.0319419.s003.docx]

We address each of the editor and reviewer comments below in bold-italics and provide our responses in blue, indicating where we felt changes in the original manuscript was warranted.

***1. Please ensure that your manuscript meets PLOS ONE's style requirements, including those for file naming. The PLOS ONE style templates can be found at***

[***https://journals.plos.org/plosone/s/file?id=wjVg/PLOSOne_formatting_sample_main_body.pdf [journals.plos.org]***](https://urldefense.com/v3/__https:/journals.plos.org/plosone/s/file?id=wjVg*PLOSOne_formatting_sample_main_body.pdf__;Lw!!PDiH4ENfjr2_Jw!F2GGfCGqKgJCRF8w22rN0hARgeAzA_Xw-PcU3jYbbN1cvva-a2DkkUhztAsJdmrgW0IEAtJ3xLljH3r7LPE8lYs3q3c$) ***and*** 
[***https://journals.plos.org/plosone/s/file?id=ba62/PLOSOne_formatting_sample_title_authors_affiliations.pdf [journals.plos.org]***](https://urldefense.com/v3/__https:/journals.plos.org/plosone/s/file?id=ba62*PLOSOne_formatting_sample_title_authors_affiliations.pdf__;Lw!!PDiH4ENfjr2_Jw!F2GGfCGqKgJCRF8w22rN0hARgeAzA_Xw-PcU3jYbbN1cvva-a2DkkUhztAsJdmrgW0IEAtJ3xLljH3r7LPE89VbdfSs$)

>> We have reviewed and followed the template guidance.

2. Thank you for stating the following financial disclosure:

***“The views expressed in this publication are those of the author(s) and not necessarily those of the NIHR or the UK Department of Health and Social Care.”***

***Please state what role the funders took in the study.  If the funders had no role, please state: "The funders had no role in study design, data collection and analysis, decision to publish, or preparation of the manuscript." If this statement is not correct you must amend it as needed.***

***Please include this amended Role of Funder statement in your cover letter; we will change the online submission form on your behalf.***

>> The revised funding statement now reads – we will also include this in our cover letter.

“This research is funded by the National Institute for Health Care Research (NIHR) (NIHR 32027), a major funder of global health research and training, using UK aid from the UK Government to support global health research. The funders had no role in study design, data collection and analysis, decision to publish, or preparation of the manuscript."

***3. Please note that funding information should not appear in the Acknowledgments section or other areas of your manuscript. We will only publish funding information present in the Funding Statement section of the online submission form. Please remove any funding-related text from the manuscript.***

>> We have removed the funding statement from the manuscript.

***4. We noted in your submission details that a portion of your manuscript may have been presented or published elsewhere:***

***“A version of this protocol is preprinted which has been uploaded.”***

***Please clarify whether this [conference proceeding or publication] was peer-reviewed and formally published. If this work was previously peer-reviewed and published, in the cover letter please provide the reason that this work does not constitute dual publication and should be included in the current manuscript.***

>> We are not really sure the context behind this query – a preprint is by definition not peer reviewed and is a posting for visibility rather than a publication. The route from MedRXiV (the preprint) to PLOS ONE is an allowable direct transfer in accordance with the guidance ([Advancing the sharing of research results for the health sciences](https://www.medrxiv.org/content/about-medrxiv)). We also mention this in the covering letter.

***5. Please include captions for your Supporting Information files at the end of your manuscript, and update any in-text citations to match accordingly. Please see our Supporting Information guidelines for more information:*** [***http://journals.plos.org/plosone/s/supporting-information [journals.plos.org]***](https://urldefense.com/v3/__http:/journals.plos.org/plosone/s/supporting-information__;!!PDiH4ENfjr2_Jw!F2GGfCGqKgJCRF8w22rN0hARgeAzA_Xw-PcU3jYbbN1cvva-a2DkkUhztAsJdmrgW0IEAtJ3xLljH3r7LPE8A1eia-c$)***.***

>> There are no supporting information files in this protocol.

***6. Please review your reference list to ensure that it is complete and correct. If you have cited papers that have been retracted, please include the rationale for doing so in the manuscript text, or remove these references and replace them with relevant current references. Any changes to the reference list should be mentioned in the rebuttal letter that accompanies your revised manuscript. If you need to cite a retracted article, indicate the article’s retracted status in the References list and also include a citation and full reference for the retraction notice.***

>> No changes to references have been made and no references have been retracted.

**Additional Editor Comments:**

Recommendations:

1. ***Required Change – Clarify Use of LMIC Terminology:
   The broad use of LMICs may obscure significant socio-economic and healthcare disparities within this group. Action needed: Narrow the scope to low-income countries (LICs) or clearly discuss the impact of regional differences on the study’s outcomes.***

>> We will remain with LMIC as we can only involve participants from our partner countries. Our lead country for this project is Zimbabwe which is listed as LMIC (not LIC) according to the 2024/2025 World bank classification. [***World Bank country classifications by income level for 2024-2025***](https://blogs.worldbank.org/en/opendata/world-bank-country-classifications-by-income-level-for-2024-2025)***.*** All our other participant countries also have LMIC status with the exception of Uganda.

Our search for potential outcomes to include in the COS comes from a variety of different research undertaken in different LMIC settings so we believe any societal or regional differences will be captured or come to light in the consensus building stages of this research. We have added a sentence on this in the protocol discussion.

2. ***Recommended Change – Address Regional Variations:***
   ***Differences in healthcare systems, socio-economic conditions, and cultural factors could influence the outcomes. Recommendation: Discuss these variations and how they may affect the outcomes identified in different LMIC settings.***

>> Thanks for this comment. The final consensus meeting which will have representation from all countries will ask participants to comment on these factors once the final COS is agreed. We can then address these in our final reporting. We’ve extended our considerations to finalising the COS in the protocol to take into account these factors.

***Reviewer #1: The study protocol presents an important justification for studies in the area of maternal and child health. Its methodology presents an aspect that should be highlighted: the inclusion of patients in the definition of outcomes. Because these patients belonged to low and medium development countries, the COS related to respectful maternal and newborn care will have a greater chance of being valid for this contexts.
The methodology is feasible and the description provide sufficient methodological detail for the protocol to be reproduced and replicated.
The authors justify the changes to the sample after the pre-print published.
The acronym for Study Steering Committee (SCC) in page 5 doesn't seem to be correct.
I have no suggestions. I believe it should be published.***

>> Thank you for acknowledging the importance of our work and for recommending publication. We have changed the acronym SCC to SSC throughout.
